# Post-TB sequelae and care: a systematic review and synthesis of qualitative research

**DOI:** 10.5588/ijtldopen.25.0084

**Published:** 2025-06-13

**Authors:** C.L. Leung, D. Jerene, J. Meghji, M. Drage, W. Mbawala, S.G. Mpagama, C. Pell, C. Mulder

**Affiliations:** ^1^ KNCV TB Foundation, The Hague, The Netherlands;; ^2^ Imperial College Healthcare NHS Trust, London, UK;; ^3^ LHL International Tuberculosis Foundation, Oslo, Norway;; ^4^ Mwitikio wa Kudhibiti Kifua Kikuu na Ukimwi Tanzania (MKUTA), Tanzania;; ^5^ Kibong’oto Infectious Diseases Hospital, Kilimanjaro, Tanzania.

**Keywords:** tuberculosis, post-tuberculosis lung disease, PTLD, pulmonary rehabilitation

## Abstract

**BACKGROUND:**

TB has long-term health and social sequelae. The experiences of TB survivors are not well understood and there is limited evidence around gaps in care. This article aims to provide a comprehensive overview of qualitative research on post-TB sequelae and care, to identify knowledge gaps and inform future research and interventions to support person-centred care.

**METHODS:**

A systematic search strategy, using two search strings incorporating post-TB and TB-related chronic respiratory disease. Searches were conducted on PubMed, Web of Science and CINAHL. Sources were screened systematically, data extracted independently and analyzed thematically.

**RESULTS:**

Sixty-six sources were identified. After applying exclusion/inclusion criteria, 16 articles were included in a qualitative synthesis. Key themes included the physical, psychological, economic and social impacts of TB. These included threats to TB survivors’ social role. People who suffer from long-term sequelae are stigmatised. Access to care is limited and tends to focus on acute respiratory disease. Policymakers indicate that the lack of data regarding the long-term impacts of TB contributed to insufficient resources being allocated.

**CONCLUSION:**

This systematic review underscores the post-TB physical and psychological impacts and the complexity of post-TB sequelae; it emphasizes the urgent need for evidence regarding the long-term impact of TB sequelae to improve care.

TB is an acute and chronic threat to global health and wellbeing. In 2022, there were an estimated 10.6 million people each year suffering from the disease and 1.3 million deaths were attributed to TB.^1^ For many people with TB disease, particularly in cases of drug-susceptible TB, treatment success is high, at around 80%.^2^ In 2020, estimates suggests that there could be over 150 million TB survivors alive.^3^ The morbidity and mortality post-TB treatment is often underappreciated.^4^ Available data indicate that around 50% of TB survivors experience long-term health problems after TB disease.^4^ For example, pulmonary TB (PTB) can have long-term consequences for survivors, particularly in terms of chronic lung disease (CLD).^4^ Respiratory sequelae from TB are compounded by environmental factors, such as indoor/outdoor air pollution and smoking, along with concurrent respiratory diseases, such as asthma.^5–7^ Post-TB morbidity can include lung disability, neurological impairment, cardiac disorders, and mental health problems.^5,8,35^ With data on the extent of post-TB sequelae often incomplete or absent, morbidity estimates likely underestimate the real burden.^9^

Poverty and TB are insidiously linked: the world’s poorest are at greatest risk of disease and disease episodes leave deep economic scars.^10,11^ Furthermore, the impacts can be social, with loss of social status linked to economic shocks and TB-related stigma,^8^ and can continue after treatment. Hence, even after eradicating TB, the ramifications of past disease episodes will persist for many decades. Despite these wide-ranging impacts, limited data are available on the experience and needs of TB survivors.^8,9^ Such data are crucial to adapt services and to ensure a person-centered approach throughout the TB care cascade.^12^ Qualitative research is a useful approach to gain insights into the experiences and needs of TB survivors and their households. Healthcare providers, TB programme staff and policymakers, have important contributions to make regarding available post-TB services and health system challenges for implementation. Drawing on a systematic search strategy and qualitative synthesis, this article maps existing qualitative research on stakeholders’ experiences of post-TB morbidity, identifies emerging themes, outlines key considerations for person-centred care post-TB and highlights priority areas for future research.

## METHODS

The PRISMA 2020 guidelines guided the systematic review and reporting of results. Two sets of search terms were used: 1) to capture experiences of post-TB morbidity; and 2) to incorporate CLD and other chronic respiratory conditions potentially caused by TB (Table 1). The databases searched included PubMed, Web of Science, and CINAHL. The search terms were also used in Google Scholar, and we reviewed citations used in the included sources. All searches were conducted on 3^rd^ December 2024. Ethical approval was not required for this systematic review and synthesis.

**Table 1.** Search terms.

Search 1: post-TB	Search 2: chronic lung disease related to TB
(post-TB OR post-Tuberculosis OR TB-sequelae OR tuberculosis-sequelae)	((CRD OR CLD OR "Chronic lung disease" OR "Chronic respiratory disease")
	AND
	(TB OR Tuberculosis))
AND	
(qualitative OR interview* OR focus-group OR ethnograph* OR participatory OR phenomolog*)	

### Eligibility criteria

We included studies focusing on post-TB which refers to chronic respiratory impairment in individuals attributed to a prior history of PTB.^29,30^ We only included studies employing qualitative research methods. The results of mixed-methods studies were included, but only data derived from qualitative methods were extracted. Only English-language publications were included. Protocols, conference papers and opinion pieces were excluded (Table 2). There were no restrictions on study location, participant age, or publication date. The screening process was completed independently by two researchers, CP and CLL, in two stages (title and abstract screening, followed by full-text review) with discussion used to resolve disagreements. Screening was completed in EndNote 21.

**Table 2.** Inclusion and exclusion criteria.

Inclusion criteria	Exclusion criteria
Empirical research	Chronic lung disease that are not linked with TB
Data collected using qualitative research methods (in-depth or semi-structured interviews; focus group discussions; participatory methods; participant observation; structured observation)	Research protocols/ review
Data collected on experiences of post-TB sequalae or care (including rehabilitation) for post-TB symptoms	Non-English language
Published in a peer-reviewed journal	Conference papers/ editorials/ opinion pieces
	Review

### Data analysis and qualitative synthesis

Two authors (CP and CLL) independently extracted data into an MS Excel spreadsheet. We then analyzed the data thematically using a combination of deductive and inductive methods to identify themes from each included source. The authors then discussed the identified themes and sub-themes and consolidated a final list (Supplementary Data). There was no major disagreement between the authors on the themes.

## RESULTS

We identified 102 studies in total (see Figure). After removing 36 duplicates, 66 remained for screening per the inclusion and exclusion criteria. Following initial screening, 30 studies were excluded, leaving 36 eligible for abstract review. After this review, 21 records were excluded, resulting in 15 studies for full-text review. Through Google Scholar, we identified one further eligible source, bringing the total to 16 references for full-text review. All studies were published between 2018 and 2024 (Table 3). Six were multi-country studies primarily conducted in Africa; seven were conducted in Africa; one in Central Asia; one in South America and one in South Asia. Common qualitative methods included interviews, focus group discussions, and observations. Eight studies focused on post-TB, and the remaining eight on CLD/CRD, where post-TB is one of the causes. Six described the experiences of people with post-TB sequelae or TB-related CLD/CRD, seven described a rehabilitation program and three examined the impact of post-TB sequelae or CLD/CRD. One study included adolescents as respondents.^13^ All included sources were based on research with a cross-sectional study design. The included studies report on the perspectives of patients, healthcare providers, community members and policymakers. We identified three overall themes related to ‘experiences of TB survivors’, ‘post-TB care’ and ‘knowledge gaps around post-TB sequelae’.

**Figure.**
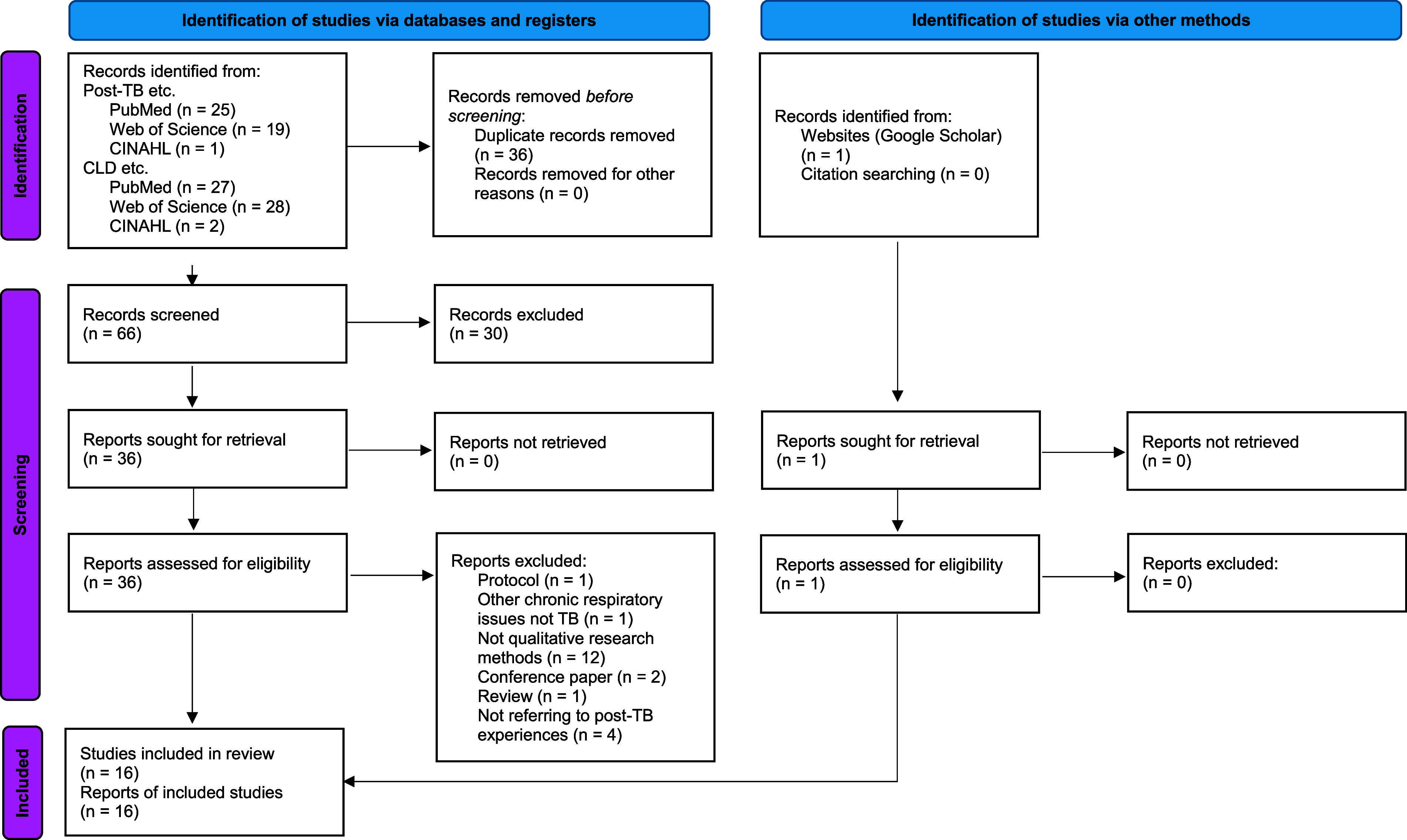
Prisma 2020 flow diagram.

**Table 3.** Overview of the included publications.

Author	Morbidity	Year	Study location(s)	Methods	Respondent type and number
Saleh S, et al.^21^	CLD/CRD	2018	Kasungu district, Central Malawi	FGDs; interviews	4 Community members;
					14 Community health actors;
					14 patients with chronic cough/TB, asthma, COPD or bronchiectasis
Jones R., et al.^14^	CLD/CRD	2018	Mulago Hospital - Outpatient department, Kampala, Uganda	Interviews; FGDs; ethnographic observations	42 patients with chronic respiratory disease
Meghji J, et al.^20^	Post-TB	2020	Urban, Blantyre, Malawi.	Interviews; questionnaire-based survey	21 people who had completed TB treatment ≥12 months
Philip KE, et al.^26^	CLD/CRD	2021	Makerere University Lung Institute outpatient clinic, Central Kampala, Uganda	Semi-structured interviews	8 Healthcare providers;
					11 clients with chronic respiratory disease
Loveday M, et al.^12^	Post-TB	2021	Umzinyathi District, KwaZulu-Natal South Africa	Interviews	12 individuals who were successfully treated for RR-TB
Egere U, et al.^23^	CLD/CRD	2021	Sudan, Tanzania	In-depth interviews	25 key informants interview (Tanzania n=12; Sudan n=13)
Wademan D, et al.^19^	Post-TB	2021	Western Cape, City of Cape, Town Metropole, South Africa; Zambia	Semi-structured interview	103 ≥18 TB patients completed treatment (South Africa n=25; Zambia n=78)
Brakema EA, et al.^22^	CLD/CRD	2022	Uganda; Kyrgyzstan (Naryn and Chui); Vietnam; Greece (Roma camp and rural areas, Heraklion region)	FGDs; semi-structured interviews; structured observations; questionnaire-based survey; document review	77 interviews;
					45 focus groups;
					61 household observations;
					22 health provider observations;
					204 health-care professional survey: (Uganda n=41, Kyrgyzstan Naryn n=42, Kyrgyzstan Chui n=40, Vietnam n=40, Greece n=41);
					1037 community survey (Uganda n=207, Kyrgyzstan Naryn n=210, Kyrgyzstan
					Chui n=210, Vietnam n=210, Greece n=200)
Mademilov M, et al.^17^	Post-TB	2022	Bishkek and Chui region, Kyrgyz Republic	Interviews; FGDs	12 pulmonologists;
					3 TB specialists;
					48 adults with PTLD
Mulupi S, et al.^24^	CLD/CRD	2022	Primary, secondary and tertiary health facilities, government agencies and civil society organisations in Kenya, Malawi, Sudan, Tanzania and Uganda	Interviews	60 national or district-level policy stakeholders;
					49 healthcare workers
Karanja S, et al.^15^	Post-TB	2022	Kenya, Malawi, international organizations	Interview, workshop discussion	38 interviews with TB-survivors, healthcare workers, funders, policy-makers and researchers. (Malawi n=12; Kenya n=20; international organizations n=6)
Nkereuwem O, et al.^13^	Post-TB	2023	The Gambia (with respondents from across West Africa)	Group discussions	10 adolescents (13–20 y/o);
					23 adult TB survivors and representatives of TB advocacy groups (21–60 y/o);
					5 policy sector participants (3 NTP officers, 1 WHO staff and 1 International NGO representative)
Egere U, et al.^16^	CLD/CRD	2023	Gezira state, Sudan; Dodoma region, Tanzania,	In-depth interviews, FGDs	12 people with known or suspected CRD;
					14 community members
Habib GM, et al.^18^	CLD/CRD	2024	Community-based PR centre, Khulna, Bangladesh	Interviews	15 clients with chronic respiratory disease
Almeida C, et al.^25^	Post-TB	2024	Lima, Peru	individual interviews, FGDs	40 intervention participants (FDGs n=15);
					4 TB program advisors;
					13 TB program staff
Meghji J, et al.^28^	Post-TB	2024	Blantyre, Malawi	In-depth interviews, FGDs	23 In-depth interviews with PTB-survivors ; FDG with 12 health care workers and 18 TB Officer

FGDs = focus group discussions; PR = pulmonary rehabilitation; CLD = chronic lung disease; CRD = chronic respiratory disease; RR-TB = rifampicin-resistant TB; COPD = chronic obstructive pulmonary disease.

### Experiences of TB survivors

Six studies described how individuals with morbidity linked to past TB disease (including CLD or CRD) experience various physical impediments, such as breathlessness, chronic cough, and fatigue, even after completing treatment.^12,14,16–19^ These issues hindered their ability to re-engage in physical activities and work,^12,13,15,19^ further harming their financial situation. For some, poverty led to malnutrition, which worsened their overall health.^20^ Included in the reviewed sources, respondents from one study reported feeling positive about completing TB treatment,^12^ whereas three publications highlighted the traumatic and long-lasting psychological effects of TB, with survivors reporting anxiety about relapse,^12,17,19^ and some blamed themselves for the ongoing symptoms.^28^ Moreover, post-TB symptoms prevented them from performing their social roles, which negatively impacted their mental health.^14,16^ Financial pressure also contributed to their negative emotions.^20^ Studies highlighted the importance of psycho-social support from communities, families and in facilities.^13,15^

Study participants described how post-TB sequelae or CLD influenced their social relationships, citing experienced^12-14,16,17,19,20^ or internalised stigma.^16,17^ Due to their symptoms, they often abstained from social interactions to avoid being stigmatized,^13,16^ with the wider community associating symptoms – particularly chronic cough – with TB or HIV.^16,20^ Stigma was experienced in various settings, including family, workplaces, communities, and care facilities.^12,17,19^ One study reported, family members of TB survivors also faced stigma.^13^ Consequently, individuals were often reticent to disclose their health status and change their way of living.^17,19^ In one study, the inability to return to work particularly affected men, who felt a loss of social standing.^20^ Post-TB sequelae or CLD often reportedly affected individuals' financial circumstances. Where the physical and mental impacts of the disease extended after treatment, survivors are often unable to return to work, especially when this entails physical exertion.^12–17,19,20^ TB treatment and prolonged post-TB recovery further added to their burden, resulting in a deterioration of their living standards and support their family.^12,13,16,20^

### Post-TB care

The care-seeking behaviour of individuals with post-TB sequelae or CLD was influenced by multiple factors. People tended to seek care promptly if experiencing acute conditions, such as chronic cough.^19,21,22^ The nature, severity and understanding of their symptoms also affected their decisions to seek care.^17,21^ A study indicated care-seeking decisions were made collectively within families and influenced by older relatives.^21^ Healthcare costs and financial situations determine whether they will seek post-TB care.^15,16^ Some individuals established trust with healthcare workers and followed their advice.^19^ Others relied on alternative methods, such as spa treatments, diet, and exercise,^17,22,28^ because they were not sure where to seek help. Care experiences varied across studies. In some settings, healthcare workers presumed that symptomatic TB survivors had TB rather than post-TB sequelae or CLD^22,28^ and provided insufficient information.^21^ One study mentioned contradictory diagnoses and advice was sometimes given by healthcare workers.^28^ They also focused on treating active PTB, with little attention for their general health and wellbeing.^13^ Several barriers to accessing pulmonary rehabilitation (PR) programs were described. These include a shortage of trained staff and a lack of clear care and referral guidelines, which affect diagnosis and reporting.^15,22–24,28^ Health systems were not prepared to provide PR programs,^15,21,24,28^ often requiring people to travel long distances to access care.^21,24^ The costs associated, including travel expenses, prevented many from participating in PR.^17,18,21,25^ Only one study describing online PR was identified, and reported that this presented challenges for elderly individuals.^25^ Two studies illustrated that PR programs for post-TB sequelae or CLD are currently unavailable in respondents’ countries due to the prioritization of other public health issues.^15,23^ People who joined rehabilitation programs generally had positive experiences, describing how programs improved their physical and mental health along with social relationships.^14,17,25,26^ Many participants continued the exercises after the program ended.^14,25^ However, some reported that the program intensified their health issues.^18^ Participants from one study emphasized the importance of helping individuals reintegrate into society by providing social protection schemes and vocational training as part of post-TB rehabilitation programs.^15^ One publication reported how participants found that the rehabilitation program provided social support and that the unspoken competition within the group motivated them to exercise harder.^18^ Study participants also described factors that could improve the acceptability and uptake of rehabilitation programs. They emphasized the importance of adapting the program to the local context,^26^ and reframing rehabilitation positively by focusing on the curability of TB.^15^ Conducting PR in a group setting helped participants stay involved and receive peer support.^26^ Increasing understanding of the rehabilitation programs can foster their positive attitudes and help manage their expectations.^18^ Additionally, healthcare workers need to be trained.^17,25^

### Knowledge gaps around post-TB sequelae

Research participants, including TB survivors and healthcare workers, acknowledged their lack of knowledge regarding post-TB sequelae, CLD and related PR.^13,15,19,21-23,28^ Participants described diverse perceptions regarding the curability of TB, with some suggesting an association between HIV and TB.^19^ Insufficient knowledge and understanding were described by TB survivors as leading to potential discrimination and hindering the development of rehabilitation programs.^14,18^ Additionally, the lack of data on CLD, inadequate policies, and limited health system capacity negatively affect the development of these programs.^24,25^ The lack of data about the conditions also serves as a barrier for additional funding.^15^

## DISCUSSION

Despite the estimated 150 million TB survivors,^3^ we identified only 16 studies that used qualitative research methods to examine experiences of sequelae and care post-TB. Furthermore, several studies focused on CLD/CRD, with TB as one potential aetiology, and not all incorporated TB survivors as respondents. The identified studies were geographically diverse, although generally conducted in high-TB burden settings, and mostly in low- or middle-income countries, with one exception (Greece).^22^ Only one study included children or adolescents^13^ – despite the high burden of TB in this age group,^27^ this was also reported in other post-TB scoping review.^31^ Although post-TB sequelae can have financial implications, further evidence is needed to determine whether these can be classified as catastrophic TB-related costs. This research highlights the paucity of evidence in this area.

Our review highlights an urgent need for further research to incorporate the long-term socioeconomic and psychological needs, experiences and impacts of TB survivors. Research that draws on qualitative research methods is crucial to ensure that the perspectives of TB survivors and other relevant actors are examined fully, with the diversity of experiences captured. Ideally, qualitative research would also take a longitudinal approach, and go beyond single interactions (on which the identified studies relied) to gain an in-depth (and longer-term) perspective on living with post-TB sequelae.^33^ The findings resonate with the quantitative evidence, highlighting that sequelae post-TB are not limited to people’s physical health;^8^ the physical impacts are entwined with psycho-social and socio-economic implications. TB can lead to long-term debilitating symptoms that are stigmatized (including chronic cough) and restrict TB survivors’ livelihoods and their capacity to fulfil the social roles that they undertook prior to their illness.^14,16^ This underlines how appropriately designed post-TB care, including PR programmes for respiratory symptoms, could have benefits beyond alleviating specific symptoms.

The reviewed studies highlight the complex presentation of post-TB lung disease (PTLD) and their overlap with the symptoms of acute TB disease, and other chronic respiratory conditions. They also illustrate how healthcare providers accustomed to managing acute TB disease tend to view some of these symptoms, such as chronic cough, through this lens and are hence less likely to consider post-TB sequelae as the underlying cause. This may be driven by limited awareness of post-TB morbidity. Hence, when seeking care, TB survivors often face an ill-equipped health system. The lack of emphasis and resources on post-TB has largely resulted from the limited data collected on post-TB sequelae, which has led to little attention from national and – until recently – international policy stakeholders.^32^ To address this, post-TB-related data should be incorporated into programmatic indicators.

The results point to the challenges in addressing the burden of post-TB sequelae. The symptoms that TB survivors report are diverse and some likely require long-term care in contexts without rehabilitation programmes for chronic respiratory symptoms. The results offer insight into the potential for success of such programmes,^34^ which were appreciated by participants. However, the chronic respiratory symptoms that TB survivors experience can have complex aetiologies: linked to determinants, such as exposure to air pollution and smoking. Although rehabilitation programmes are an important component in addressing post-TB sequelae, they may be insufficient on their own to fully address these issues.

The review is strengthened by the use of two search strategies to incorporate additional research on CLD or CRD that likely resulted from TB (but not explicitly termed post-TB). Further strengths include the use of multiple databases, Google Scholar and bibliographic searching. The conclusions that can be drawn in this review are constrained by the studies under review, and their weaknesses, with many relying on single data collection methods, single respondent types and limited to research available in English.

## CONCLUSION

This systematic review and synthesis of qualitative research on experience of post-TB sequelae and care highlights a paucity of research in this area. Sixteen articles, published between 2018–2024, were identified across diverse geographic locations. The qualitative synthesis highlighted the intertwined and long-term health, social and economic impacts of TB disease. Appropriate care for PTLD was limited, with TB survivors facing barriers to care, linked to the lack of familiarity with TB sequelae among health staff and a focus on acute TB disease. Available rehabilitation programs were well received. There are clear evidence gaps regarding post-TB experiences. Further qualitative research that uses longer-term approaches and that incorporates TB survivors, particularly adolescents and children who have experienced TB disease, is urgently needed.
